# Trends and age-period-cohort effect on incidence of hepatitis B from 2008 to 2022 in Guangzhou, China

**DOI:** 10.1038/s41598-024-63796-0

**Published:** 2024-06-11

**Authors:** Zhiwei Zheng, Xinqi Lin, Yong Huang, Chunhuan Zhang, Zhoubin Zhang

**Affiliations:** 1 Guangzhou Center for Disease Control and Prevention, Guangzhou, 510440 China; 2 Institute of Public Health, Guangzhou Medical University & Guangzhou Center for Disease Control and Prevention, Guangzhou, 510440 China

**Keywords:** Viral hepatitis, Risk factors

## Abstract

Hepatitis B virus (HBV) infection is highly prevalent in Guangzhou, China. This study aimed to examine the long-term trend of HB incidence from 2008 to 2022 and the independent impacts of age, period, and cohort on the trends. HBV data were collected from the China Information System for Disease Control and Prevention. Joinpoint regression was utilized to examine temporal trends, and an age-period-cohort model was employed to estimate the effects of age, period, and cohort. A total of 327,585 HBV cases were included in this study. The incidence of chronic and acute HB showed a decreasing trend in Guangzhou over the past 15 years, with an average annual percent change of − 4.31% and − 16.87%, respectively. Age, period, and cohort all exerted significant effects. The incidence of HB was higher in males than in females and non-central areas compared to central areas. Age groups of 0–4 years and 15–24 years were identified as high-risk groups. The period relative risks for chronic HB incidence decreased initially and then stabilized. Cohorts born later had lower risks. Chronic HB incidences remain high in Guangzhou, especially among males, younger individuals, and residents of non-central areas. More efforts are still needed to achieve hepatitis elimination targets.

## Introduction

Hepatitis B virus (HBV) is a serious global public health concern, particularly in underdeveloped countries^1^. HBV infection can lead to acute and chronic hepatitis, and ultimately develop into life-threatening complications such as liver cancer or cirrhosis. The World Health Organization (WHO) estimated that hepatitis B caused 820 thousand deaths in 2019, with 296 million people living with chronic HBV infection and 1.5 million new infections each year^2^. Therefore, the WHO has set the goal of eliminating hepatitis B as a public health threat by 2030, with the target of reducing new infections by 90% and mortality by 65%^3^.

The prevalence of HBV infection varies geographically, and regions can be broadly classified into high (> 8% hepatitis B surface antigen (HBsAg) prevalence), higher-intermediate (5–7.99%), lower-intermediate (2–4.99%), and low (< 2%) prevalence areas^4^. According to this criterion, China has transitioned from a high-prevalence area to a higher-intermediate prevalence area. The weighted prevalence of HBsAg adjusted for the population aged 1–59 years decreased from 9.8% in 1992 to 7.2% in 2006 and further declined to 6.1% in 2016^5,6^. However, China still has approximately 70 million HBV infections, suggesting that China has the world’s heaviest burden of HBV infection and will make a significant contribution to the global elimination of hepatitis B by 2030^1,7^. Efforts are still needed to minimize HBV transmission in the country.

Guangzhou, located in southeast China, has the highest HBV prevalence nationwide. The weighted prevalence of HBsAg in Guangzhou decreased from 12.45% in 2008 to 9.50% in 2018. However, the prevalence in Guangzhou was still substantially higher than the national average and the city remains a high prevalence area^6,8^. Specifically, some high-risk populations deserve attention to control HBV infection in this city, such as large-scale migrants and those living in economically underdeveloped areas. In 2020, nearly 9.4 million labor workers migrated to Guangzhou in search of better opportunities, and migrants from areas with low to moderate prevalence are susceptible to HBV infection in this high-prevalence city. Unfortunately, little research has investigated the changing trends of hepatitis B infection in Guangzhou.

Previous research has found that hepatitis B is subject to different temporal trends. It may vary depending on age, period, and birth cohort^9,10^. For example, due to sexual transmission, adults aged 35–54 are more susceptible to HBV infection^11^. With the universal vaccination of newborns, the global HBV infection rate has been decreasing in recent decades. From 2008 to 2019, the HBV incidence among US blood donors decreased from 3.4 to 2.4 per 100,000 person-years, representing a 29% reduction^12,13^. 90% of newborns and infants infected with hepatitis B will develop chronic infection, demonstrating that the age of infection is a key determinant of chronic infection, and the birth cohort may be a crucial factor^14^. Furthermore, differences in hepatitis B prevalence are frequently found between women and men, as well as among different regions^9,10^. When evaluating temporal trends, it is important to note that age, period, and cohort effects are highly correlated with each other. Given the changes in risk factors and demographic characteristics over time, the risk of HBV infection may vary between different birth cohorts^15^. Overall, there is limited research on the use of the age-period-cohort model (APC model) in HBV-related trend analysis^9,16^. Understanding the patterns and causes of HBV incidence trends is crucial for designing more effective prevention strategies. Therefore, the current study aims to use the APC model to assess the effects of age, period, and birth cohort on HBV incidence trends among different genders and regions, and to provide evidence for effective prevention and control of hepatitis B in a high-prevalence area.

## Materials and methods

### Data source

Two datasets were used in this study. The data on HBV infection during 2008–2022 in Guangzhou were obtained from the China Information System for Disease Control and Prevention. According to the diagnostic criteria for viral hepatitis B (WS 299-2008), the diagnosis of acute and chronic hepatitis B requires a comprehensive judgment based on the symptoms, signs, epidemiology, and laboratory test results of hepatitis B. Age, sex, region, diagnosis type, diagnosis time, and other demographic and clinical characteristics are included in the case table. Annual population statistics for Guangzhou were extracted from the Guangzhou Statistical Yearbook 2008–2022. This study has been approved by the ethics committee of the Guangzhou Center for Disease Control and Prevention. Participants were informed about the study objectives, and written informed consents and assents were obtained from all participants. All study procedures were performed in accordance with the ethical standards of the institutional research committee on human experimentation.

### Statistical analysis

The eleven districts of Guangzhou were divided into two regions (central areas and non-central areas) based on factors such as geographical location and level of economic development. The central areas included Tianhe, Yuexiu, Liwan, and Haizhu districts, while the non-central areas included Panyu, Baiyun, Huangpu, Nansha, Conghua, Zengchen, and Huadu districts.

The crude incidence rate (CIR) refers to the actual incidence among all age groups, whereas the age-standardized incidence rate (ASIR) was calculated based on the world age-standardization population^17^.

A joinpoint regression model was applied to estimate the trends of HBV incidence from 2008 to 2022, using the Joinpoint Regression Program (Version 4.9.1.0. -April 2022, available through the Surveillance Research Program of the United States National Cancer Institute)^18^. Using a Monte Carlo Permutation approach, significant joinpoints can be detected, longitudinal fluctuations can be partitioned into various segments, and segment trends with statistical significance can be identified^18^. The annual percentage change (APC) and its 95% confidence interval (95% CI) were calculated for each period after fitting the natural logarithm of the incidence rate for different segments. The global trend was calculated using the average annual percent change (AAPC). APC and AAPC were considered statistically significant if their 95% CI did not overlap or *P* < 0.05 compared to the null hypothesis of having no variation. APC > 0 indicates an increasing trend in HBV incidence in the current period, while APC < 0 indicates a decreasing trend. Similarly, AAPC > 0 indicates an increasing trend in HBV incidence over the whole period, whereas AAPC < 0 indicates a decreasing trend over the entire period.

Based on the Age-Period-Cohort Web Tool (Biostatistics Branch, National Cancer Institute, Bethesda, MD, USA; https://analysistools.cancer.gov/apc/ (accessed on March 2023)), an APC model was applied to evaluate the impact of age, period, and cohort on health outcomes, which can control or eliminate the interaction between age, cohort, and other covariates^19^. The age effect refers to the differences in HBV incidence across age groups caused by factors related to aging. The period effect refers to the impact of human factors on HBV incidence, such as the development of diagnostic tests and the availability of hepatitis B vaccinations. The cohort effect refers to the change in HBV incidence due to different exposures to risk factors among people born in different years^20^. Age and period were divided into 5-year continuous intervals from 0 to 84 years, and from 2008 to 2022, respectively. Nineteen birth cohorts were summarized from 1928 to 1932 through 2018–2022. The intrinsic estimator method was integrated into the APC model to estimate the net effects for three dimensions. Since the hepatitis B vaccine (HepB) was included in the neonatal immunization program in China in 1992 and WHO’s elimination goal for viral hepatitis was declared in 2016, we took the cohort (1988–1992), period (2008–2012) and age group (20–24 years) as reference groups. We calculated the net drift (equivalent to the AAPC in joinpoint analysis), local drifts, longitudinal age-specific rates, period rate ratios (RR), and cohort rate ratios (RR). Wald chi-squared tests were used to determine the significance of the above parameters. Net drift indicates the overall annual percentage change of the expected age-adjusted rates over time. Local drifts represent the annual percentage change of the expected age-specific rates over time. Longitudinal age-specific rates are expected age-specific rates in the reference cohort, adjusted for period effects. The cohort (or period) rate ratios represent the cohort (or period) relative risk adjusted for age and nonlinear period (or cohort) effects in a cohort (or period) versus the reference one^19^. Since the period and age intervals in the APC model should be fixed and equal, and all individuals aged 85 years and above were recorded as one age group in the Guangzhou Statistical Yearbook, HBV cases aged 85 years and over were excluded from the analysis.

Descriptive analysis and plotting were performed using R 4.2.2 software. Rates between different groups were compared using the t-test or the Mann–Whitney U test according to the data distribution. Statistical significance was considered when a two-sided *P* value was ≤ 0.05.

## Results

### Overall description of hepatitis B incidence

**Figure 1:**
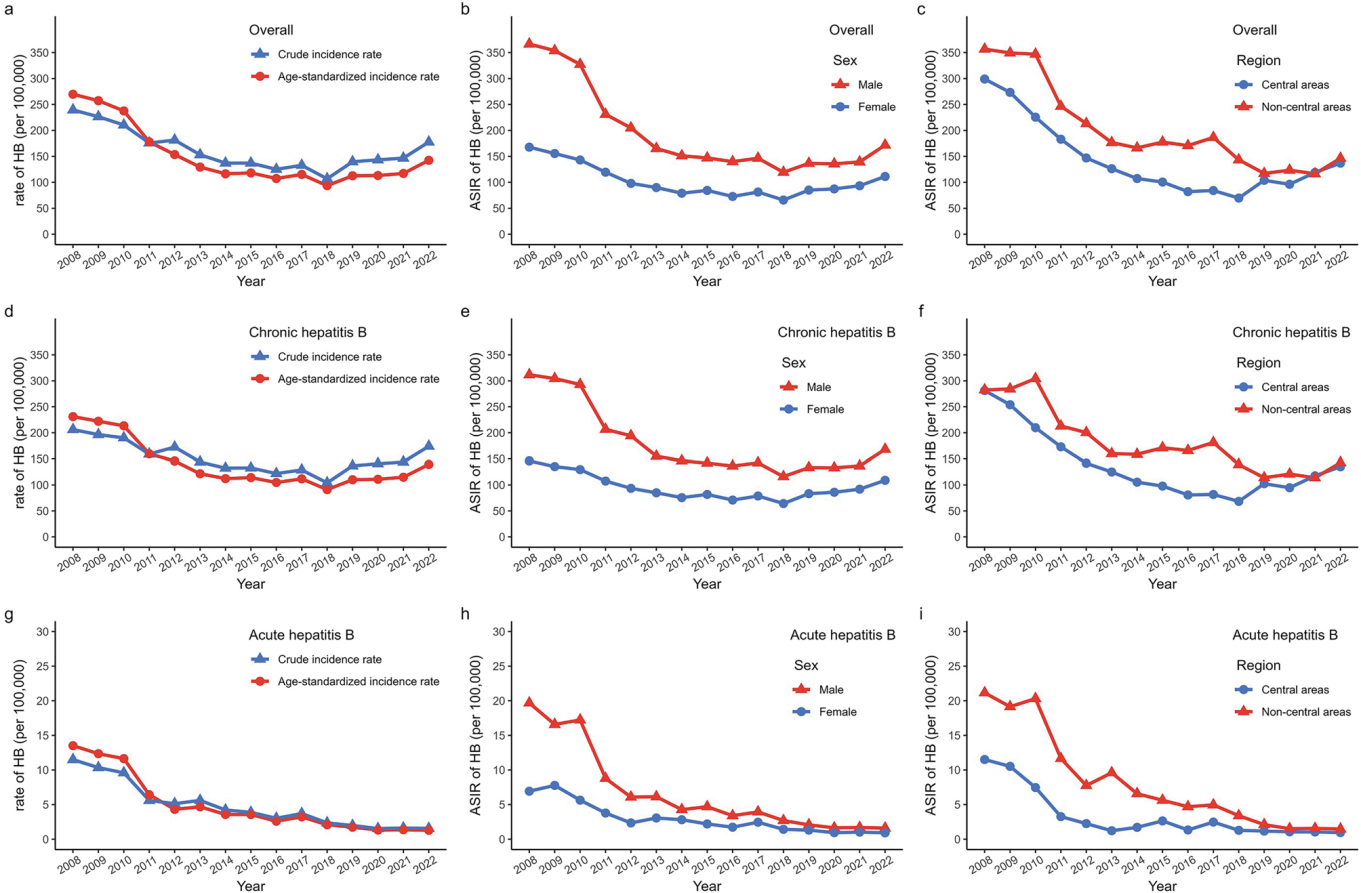
Trends of the incidence rate of hepatitis B (HB) overall and by diagnosis types in Guangzhou, China during 2008–2022. (**a**, **d**, **g**) The comparison of crude incidence rate (CIR) and age-standardized incidence rate (ASIR) of HB. (**b**, **e**, **h**) The comparison of ASIR by sex. (**c**, **f**, **i**) The comparison of ASIR by region.

As displayed in Supplementary Table S1, a total of 327,585 HBV cases were reported in Guangzhou from 2008 to 2022, with an average CIR of 162.18/100,000 and an average ASIR of 150.77/100,000. The overall trend is shown in Fig. 1a. 94.46% of the cases were diagnosed with chronic HB, 2.73% with acute HB, and 2.81% with unclassified HB. In 2022, the ASIRs for chronic and acute HB were 139.48/100,000 and 1.28/100,000, respectively. Figure 1d,g demonstrated that the trends in ASIR for chronic and acute HB were considerably different.

The overall trends by sex and by region are shown in Fig. 1b,c. Figure 1e,h showed that the ASIRs for chronic and acute HB were nearly two-fold higher in males than in females (*P* < 0.001 for chronic HB and *P* = 0.036 for acute HB), respectively. Furthermore, Fig. 1f,i demonstrated that the ASIRs for chronic and acute HB in non-central areas were higher than in central areas (*P* = 0.019 for chronic HB and *P* = 0.009 for acute HB, Supplementary Table S1).

### Temporal trends of chronic and acute HB incidence using a joinpoint regression model

**Figure 2:**
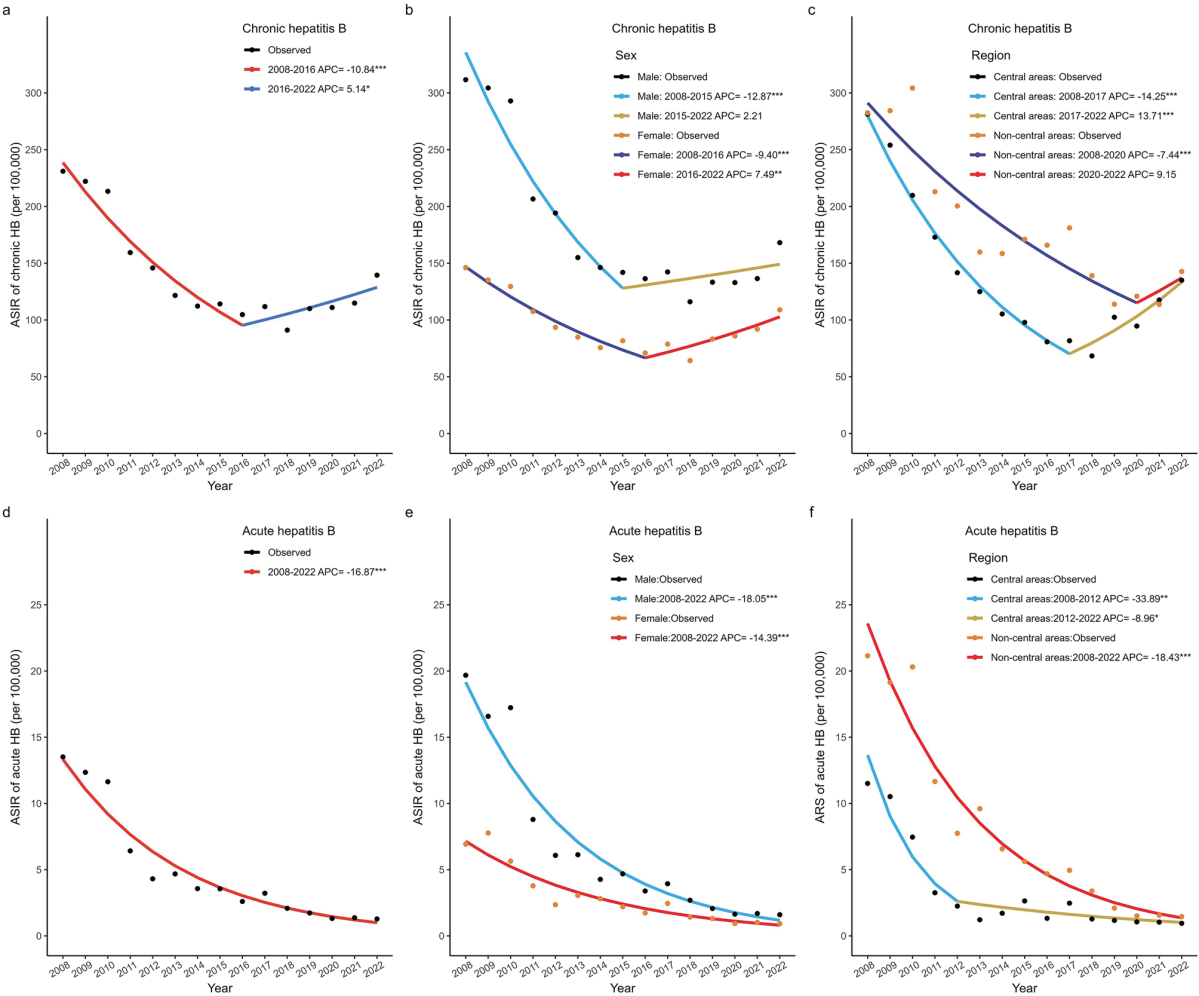
Annual percent change (APC) of hepatitis B (HB) incidence rate by diagnosis types in Guangzhou during 2008–2022, using a joinpoint regression model. (**a**, **d**) APCs of the age-standardized incidence rate (ASIR) for chronic and acute HB. (**b**, **e**) APCs for chronic and acute HB by sex. (**c**, **f**) APCs for chronic and acute HB by region.

As shown in Supplementary Table S2 and Fig. 2a–c, AAPC in chronic HB was − 4.31% (95% CI − 6.51% to − 2.06%, *P* < 0.001) over the entire study period. However, the trend of chronic HB incidence exhibited a statistically significant joinpoint from 2008 to 2022, showing two distinct trends. From 2008 to 2016, there was a significantly declining trend, with an APC rate of − 10.84% (95% CI − 13.60% to − 7.99%, *P* < 0.001). From 2016 to 2022, there was a significant increase, with an APC rate of 5.14% (95% CI 0.51% to 9.99%, *P* = 0.03). This trend was also observed in females and those living in central areas. However, the incidence rate remained relatively stable after 2015 in males and after 2020 among the people living in non-central areas (both *P* > 0.05).

Figure 2d–f and Supplementary Table S2 showed that no significant joinpoints were detected for acute HB, and the AAPC was − 16.87% (95% CI − 19.02% to − 14.65%, *P* < 0.001), which was lower than the value for chronic HB. Similarly, it also indicated a downward trend across sex and region. Particularly, the incidence rate among individuals living in central areas decreased rapidly from 2008 to 2012 (APC = − 33.89%, 95% CI − 47.91% to − 16.10%, *P* < 0.01) and then decreased relatively slowly from 2012 to 2022 (APC = − 8.96%, 95% CI − 17.02% to − 0.12%, *P* = 0.048 < 0.05).

### Age, period, and cohort effects of HB incidence using the age-period-cohort model

From 2008 to 2022, the net drift in chronic HB was − 3.76% (95% CI − 5.96% to − 1.51%, *P* < 0.01) per year (Table 1). Additionally, there were relatively significant improvements in the incidence among males and in different regions. Supplementary Figure S1–2 showed that the incidence rate was higher in the age group of 20–39 years, but the decline was more pronounced. Overall, the local drifts in the age group of 20–39 years were all below zero. The age group of 25–29 years showed the most significant improvement (local drift = − 11.46%, 95% CI − 14.34% to − 8.49%). However, Supplementary Figure S2 showed that over time, the chronic incidence rates increased in the age group of 55–59 years, and the local drift values peaked around ages 55–59 years (6.04%, 95% CI 1.24% to 11.06%), suggesting a worsening situation.

**Table 1:** The net drifts, local drifts, and statistical parameters for overall, and by sex, region using age-period-cohort models during 2008–2022 in Guangzhou, China.

Variable	Chronic HB					Acute HB				
	Overall	Gender stratification		Regional stratification		Overall	Gender stratification		Regional stratification	
		Male	Female	Central areas	Non-central areas		Male	Female	Central areas	Non-central areas
Net drifts (95% CI)	− **3.76(**− **5.96,**− 1.51)*	− **4.93(**− **7.14,**− 2.67)*	− 1.64(− 3.87,0.65)	− **5.48(**− **8.01,**− 2.87)*	− **5.24(**− **7.69,**− 2.72)*	− **12.02(**− **14.15,**− 9.84)*	− **13.31(**− **15.31,**− 11.26)*	− **9.27(**− **11.84,**− 6.63)*	− **12.64(**− **15.74,**− 9.42)*	− **14.38(**− **16.52,**− 12.19)*
Local drifts (95% CI) of age group										
0–4	− 14.52(− 36.95,15.90)	− 15.59(− 38.64,16.12)	− 12.93(− 34.55,15.82)	− 15.44(− 42.81,25.01)	− 17.04(− 39.89,14.50)	− **19.05(**− **28.07,**− 8.91)*	− **19.26(**− **27.70,**− 9.84)*	− **19.01(**− **29.42,**− 7.05)*	− **21.24(**− **34.14,**− 5.82)*	− **21.07(**− **29.85,**− 11.19)*
5–9	− 7.67(− 28.97,20.02)	− 10.23(− 31.80,18.17)	− 3.71(− 24.98,23.61)	− 10.38(− 35.85,25.20)	− 9.24(− 31.34,19.99)	− 4.29(− 18.63,12.58)	− 6.88(− 21.25,10.12)	− 0.74(− 15.65,16.81)	− 15.17(− 35.71,11.94)	− 2.72(− 17.47,14.67)
10–14	− 10.44(− 29.04,13.03)	− 11.76(− 30.20,11.56)	− 8.14(− 27.32,16.11)	− 13.62(− 35.53,15.74)	− 11.16(− 30.81,14.07)	− 8.06(− 21.54,7.74)	− 10.29(− 23.50,5.19)	− 4.42(− 19.27,13.17)	− 12.54(− 33.72,15.41)	− 8.28(− 21.77,7.53)
15–19	− 9.86(− 19.82,1.33)	− 11.23(− 21.38,0.24)	− 7.62(− 17.39,3.30)	− 11.46(− 23.51,2.48)	− 11.32(− 21.79,0.55)	− **20.49(**− **29.07,**− 10.87)*	− **21.90(**− **30.20,**− 12.61)*	− **18.05(**− **27.52,**− 7.34)*	− **18.07(**− **29.48,**− 4.82)*	− **23.34(**− **32.10,**− 13.45)*
20–24	− **10.42(**− **14.64,**− 6.00)*	− **12.6(**− **17.05,**− 7.91)*	− **7.31(**− **11.23,**− 3.22)*	− **10.6(**− **15.43,**− 5.48)*	− **12.57(**− **17.11,**− 7.77)*	− **21.43(**− **25.20,**− 17.48)*	− **22.57(**− **26.25,**− 18.71)*	− **19.74(**− **23.81,**− 15.45)*	− **20.25(**− **26.21,**− 13.82)*	− **23.90(**− **27.51,**− 20.12)*
25–29	− **11.46(**− **14.34,**− 8.49)*	− **13.12(**− **16.08,**− 10.05)*	− **8.86(**− **11.64,**− 5.98)*	− **13.16(**− **16.37,**− 9.83)*	− **12.96(**− **16.11,**− 9.69)*	− **21.60(**− **24.34,**− 18.76)*	− **22.73(**− **25.27,**− 20.09)*	− **19.23(**− **22.49,**− 15.83)*	− **20.71(**− **24.77,**− 16.44)*	− **24.10(**− **26.77,**− 21.33)*
30–34	− **8.06(**− **10.91,**− 5.12)*	− **9.27(**− **12.08,**− 6.37)*	− **5.56(**− **8.59,**− 2.44)*	− **11.03(**− **14.18,**− 7.77)*	− **9.09(**− **12.24,**− 5.82)*	− **18.26(**− **21.2,**− 15.21)*	− **19.89(**− **22.49,**− 17.19)*	− **13.63(**− **17.65,**− 9.42)*	− **18.37(**− **22.82,**− 13.65)*	− **20.66(**− **23.49,**− 17.71)*
35–39	− **5.56(**− **8.67,**− 2.34)*	− **7.13(**− **10.1,**− 4.07)*	− 2.18(− 5.73,1.50)	− **7.48(**− **10.96,**− 3.86)*	− **7.65(**− **11.05,**− 4.12)*	− **16.56(**− **19.94,**− 13.05)*	− **17.83(**− **20.77,**− 14.77)*	− **13.01(**− **17.79,**− 7.95)*	− **16.32(**− **21.56,**− 10.72)*	− **19.79(**− **23.00,**− 16.45)*
40–44	− 2.38(− 5.94,1.30)	− **3.78(**− **7.13,**− 0.31)*	0.87(− 3.31,5.22)	− 2.84(− 6.76,1.25)	− **5.56(**− **9.46,**− 1.49)*	− **13.92(**− **17.88,**− 9.76)*	− **15.05(**− **18.43,**− 11.54)*	− **10.14(**− **16.2,**− 3.64)*	− **14.05(**− **20.34,**− 7.26)*	− **17.80(**− **21.51,**− 13.91)*
45–49	− 0.50(− 4.36,3.51)	− 1.67(− 5.35,2.15)	1.99(− 2.39,6.57)	− 2.07(− 6.09,2.13)	− 2.78(− 7.22,1.87)	− **10.85(**− **15.43,**− 6.03)*	− **11.70(**− **15.69,**− 7.52)*	− **8.12(**− **14.66,**− 1.07)*	− **12.61(**− **19.62,**− 4.99)*	− **14.04(**− **18.42,**− 9.42)*
50–54	3.11(− 1.23,7.65)	1.98(− 2.27,6.41)	4.83(0.22,9.65)*	− 0.35(− 4.54,4.03)	3.20(− 2.20,8.89)	− 4.51(− 10.22,1.56)	− **5.63(**− **10.72,**− 0.26)*	− 2.92(− 10.37,5.14)	− 8.09(− 16.09,0.68)	− 5.58(− 11.31,0.53)
55–59	6.04(1.24,11.06)*	5.67(0.87,10.69)*	6.58(1.71,11.67)*	3.83(− 0.67,8.53)	5.87(− 0.18,12.29)	− 0.32(− 6.94,6.78)	− 1.11(− 7.24,5.44)	1.62(− 6.32,10.23)	− 1.09(− 9.80,8.46)	− 1.68(− 8.55,5.70)
60–64	3.03(− 2.33,8.68)	2.89(− 2.53,8.61)	3.50(− 1.83,9.12)	3.03(− 2.02,8.35)	0.69(− 5.95,7.79)	− 5.23(− 12.55,2.71)	− 7.00(− 13.81,0.35)	− 1.72(− 10.64,8.08)	− 3.71(− 13.12,6.73)	− **8.39(**− **15.94,**− 0.16)*
65–69	1.16(− 5.04,7.76)	0.89(− 5.43,7.63)	1.81(− 4.27,8.29)	1.26(− 4.54,7.43)	− 1.50(− 9.22,6.87)	− 5.55(− 15.10,5.08)	− 6.34(− 15.43,3.72)	− 3.83(− 14.78,8.54)	− 1.59(− 14.53,13.31)	− 10.39(− 19.94,0.30)
70–74	− 1.02(− 8.24,6.76)	− 1.65(− 9.03,6.33)	− 0.04(− 7.08,7.53)	− 3.20(− 9.63,3.69)	− 0.94(− 10.58,9.74)	− 6.72(− 17.43,5.38)	− 8.18(− 18.26,3.14)	− 4.65(− 17.01,9.55)	− 8.64(− 22.17,7.25)	− 7.83(− 19.19,5.13)
75–79	− 2.92(− 11.85,6.92)	− 3.21(− 12.69,7.31)	− 2.49(− 10.70,6.47)	− 6.21(− 13.97,2.25)	− 1.50(− 13.72,12.45)	− 7.25(− 19.82,7.28)	− 9.97(− 22.36,4.41)	− 3.54(− 16.89,11.95)	− 14.09(− 28.65,3.44)	− 4.01(− 18.70,13.33)
80–84	− 1.76(− 15.67,14.44)	− 2.37(− 17.40,15.39)	− 1.14(− 13.57,13.07)	− 3.55(− 15.40,9.95)	− 1.33(− 20.71,22.78)	− 5.47(− 22.86,15.83)	− 4.45(− 21.85,16.82)	− 7.61(− 25.50,14.59)	− 2.78(− 22.68,22.25)	− 8.88(− 28.22,15.66)

HB Hepatitis B; 95% CI 95% confidence interval; Net drifts represent the annual percentage change in the incidence rate based on period and birth cohort. Local drifts indicate the annual percentage change over time specific to the age group.* indicates the statistically significant (*P* < 0.05).

Significant values are in bold.

**Figure 3:**
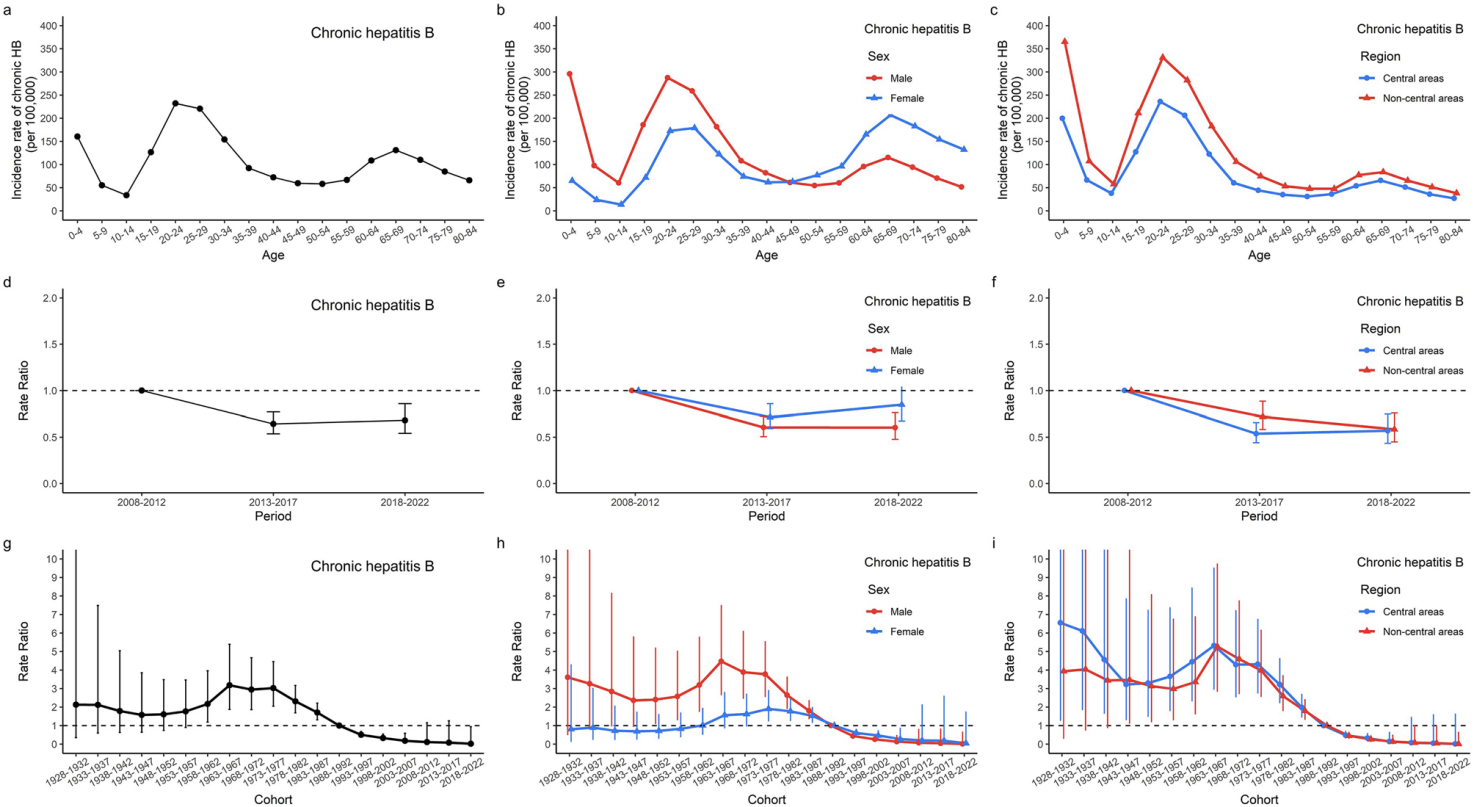
The age, period, and cohort effects on chronic HB incidence by sex and by region; (**a**, **b**, **c**) Longitudinal age curves. Longitudinal age-specific rates are expected age-specific rates in the reference cohort (1988–1992), adjusted for period effects; (**d**, **e**, **f**) Period rate ratios (RRs) and the corresponding 95% confidence intervals. The RRs of each period compared with the reference period (2008–2012) adjusted for age and non-linear cohort effects. (**g**, **h**, **i**) Cohort rate ratios (RRs) and the corresponding 95% confidence intervals. The RRs of each cohort compared with the reference cohorts (1988–1992) adjusted for age and non-linear period effects.

Supplementary Figure S1a–c showed that the age-specific chronic incidence rates have two peaks for the age groups of 25–34 years and 65–69 years. Supplementary Figure S2a showed that the chronic hepatitis B incidence rate decreased over time for children (0–4 years) and younger adults (20–24 years). However, after controlling for period and cohort effects, children (0–4 years) and younger adults (20–24 years) are at the highest risk in the longitudinal age curves of chronic incidence (Fig. 3a–c). Similar patterns in age effects were observed across sex and region. In most age groups, the chronic incidence among males was higher than among females (*P* = 0.001), but there were no significant differences between regions (*P* = 0.17).

Overall, the estimated period effects in chronic HB first decreased and then became relative stable (*RR*_*period*(2008–2012)_ = 1.00; *RR*_*period*(2013–2017)_ = 0.64, 95% CI 0.54–0.77; *RR*_*period*(2018–2022)_ = 0.68, 95% CI 0.54–0.86) (Fig. 3d). Similar patterns of period effects were observed across sex and region (Fig. 3e–f).

Cohort effects in chronic HB showed that compared to the cohort from 1988 to 1992, the cohort from 1963 to 1967 had the highest risk (*RR* = 3.18, 95% CI 1.87–5.40) and cohorts born after 1993 had lower risks. Cohorts born after 1973 experienced a faster decline, continuing to decline in the most recent cohorts (*RR*_*cohort*(2018–2022)_ = 0.02, 95% CI 0.00–0.94) (Fig. 3g–i and Supplementary Figure S4). For males and residents of non-central areas, cohorts born after 1993 had lower risks. For females and residents of central areas, cohorts from 1993 to 2007 had lower risks.

The net drift in acute HB (− 12.02%, 95% CI − 14.15% to − 9.84%, *P* < 0.001) was greater than that in chronic HB. Similarly, improvements were observed in acute incidence by sex and region. Supplementary Figure S3 demonstrated that the acute incidence rates decreased over time in most younger groups. The local drifts in the age groups of 0–4 years and 15–49 years were all below zero, indicating improvements in acute incidence. The age group of 25–29 years showed the most significant improvement (local drift = − 21.60%, 95% CI − 24.34% to − 18.76%). The local drifts by sex and region were similar to the overall trends in chronic and acute HB.

**Figure 4:**
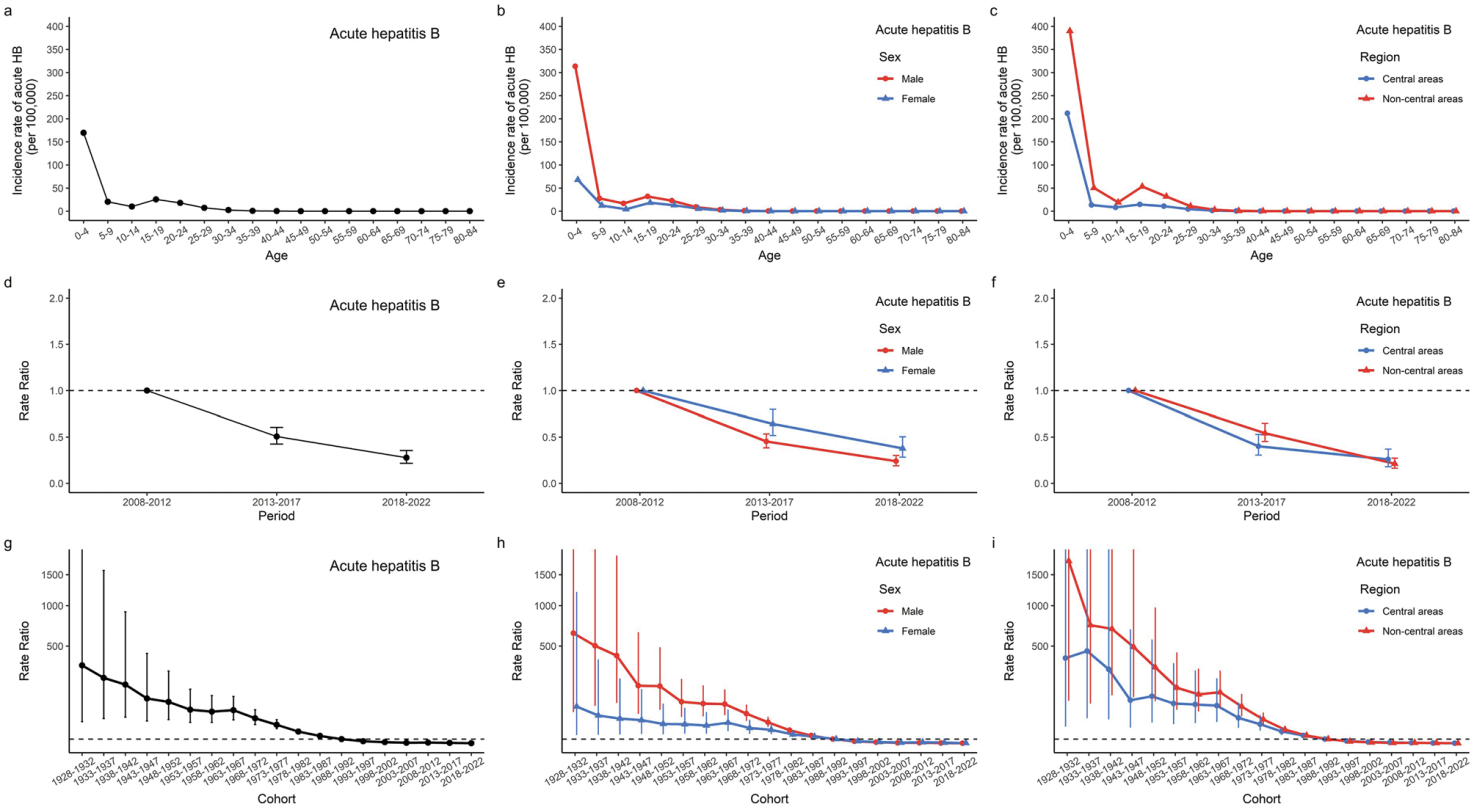
The age, period, and cohort effects on acute HB incidence by sex and by region; (**a**, **b**, **c**) Longitudinal age curves. Longitudinal age-specific rates are expected age-specific rates in the reference cohort (1988–1992), adjusted for period effects; (**d**, **e**, **f**) Period rate ratios (RRs) and the corresponding 95% confidence intervals. The RRs of each period compared with the reference period (2008–2012) adjusted for age and non-linear cohort effects. (**g**, **h**, **i**) Cohort rate ratios (RRs) and the corresponding 95% confidence intervals. The RRs of each cohort compared with the reference cohorts (1988–1992) adjusted for age and non-linear period effects.

Supplementary Figure S1d–f reveals that the age-specific acute incidence rates have two peaks at the age group of 0–4 years and 25–34 years. After controlling for period and cohort effects, children (0–4 years) and younger adults (15–19 years) are at the highest risk in the longitudinal age curves of acute incidence (Fig. 4a–c). Similar age patterns were observed across sex and region. There were no significant differences between sex (*P* = 0.63) and regions (*P* = 0.38).

The estimated period effects showed a decreasing trend in acute HB (*RR*_*period*(2008–2012)_ = 1.00; *RR*_*period*(2013–2017)_ = 0.51, 95% CI 0.42–0.60; *RR*_*period*(2018–2022)_ = 0.28, 95% CI 0.22–0.35) (Fig. 4d–f). Cohort effects in acute HB showed that the risk was lower with later birth years (Fig. 4g–i, Supplementary Figure S5). Compared with the cohort from 1988–1992, the cohort from 1928–1932 had the highest risk (*RR* = 321.04, 95% CI 25.12–4102.61), while the cohort from 2018–2022 had the lowest risk (*RR* = 0.005, 95% CI 0.00–0.03). Similar patterns of period effects and cohort effects in acute HB were observed across sex and regions.

## Discussion

Based on nearly 15 years of hepatitis B case data in Guangzhou, we performed a joinpoint analysis and an age-period-cohort analysis to elucidate the age, period, and birth cohort effects on HB incidence, while examining the influences of sex and region.

Consistent with previous research, our findings revealed significant period effects^9–11,21^. The incidence of acute hepatitis B decreased significantly over time, while that of chronic hepatitis B remained stable in recent years. Overall, the ASIR of acute and chronic hepatitis B decreased by 12.02% and 3.76%, respectively, from 2008 to 2022 in Guangzhou. The incidence of HB also decreased significantly in China and globally^10,21^. Period effects typically indicate changes directly impacting the incidence of HB, such as varying screening strategies, new diagnostic methods, changes in disease registration, and improved medical interventions. We attribute this to hepatitis B vaccination coverage and prevention of mother-to-child transmission of hepatitis B virus^22^. For instance, according to laboratory surveillance data in England, the HB incidence decreased from 7.4/100,000 in 1995–2000 to 1.1/100,000 in 2008, representing an 85% reduction^11^. In China, the plasma-derived hepatitis B vaccine (HepB) was licensed in 1985. In 1992, a recombinant vaccine was licensed, and HepB was included in the neonatal immunization program. In 2002, HepB was integrated into the Expanded Program on Immunization, providing free vaccination for children under 14. During 2009–2011, a HepB catch-up campaign was conducted in China, covering 68 million children under 15 born between 1994 and 2001. In 2011, China launched an integrated program for preventing mother-to-child transmission of HIV, syphilis, and HBV in 1156 counties (including Guangzhou), expanding it nationwide in 2015 to cover all pregnancies. The coverage of three doses of hepatitis B vaccine for infants increased from 30.0% in 1992 to 99.4% in 2019 in China^23^. Consequently, the weighted prevalence of HBsAg in individuals aged 1–59 years declined from 9.8% in 1992 to 7.2% in 2006 and further to 6.1% in 2016^5,6^.

Age is one of the most important risk factors for HB. From the perspective of age trend changes, the incidence of HB initially decreased then increased, and eventually decreased with age. After controlling period and cohort effects, we found that the age groups of 0–4 years and 15–24 years were high-risk populations. The high incidence of HBV in children aged 0–4 years might be associated with mother-to-child transmission^14,24^. It could be explained by some unhealthy lifestyle habits among young people, such as unprotected sexual behaviors between young men and women, multiple sexual partners, close contact, excessive stress among young people, frequent staying up late, drinking, and smoking^9,25^. Previous studies have suggested that administering the hepatitis B vaccine, especially to young adults at risk of HBV infection, can provide economic benefits^11,25,26^.

In addition, significant cohort effects were found. Overall, the risk of HB incidence decreased across the study cohorts, especially for acute HB^21^. People born before 1993 had a higher risk of HB compared to those born after 1993, which is probably due to the widespread use of HB vaccination^1,22^. This pattern of change in birth cohorts might also be attributed to higher levels of medical treatment, increased public awareness of hepatitis B prevention, and better living and dietary conditions^10,22^.

The incidence of HB in males was higher than in females, which is consistent with previous research^9,11^. Physiological differences between sexes might be a factor^9,11^. The incidence of HB in non-central areas was higher than that in central areas. This difference might be due to lower rates of in-hospital deliveries and mother-to-infant transmission prevention in non-central areas^4^. For example, timely birth dose coverage of hepatitis B vaccine in undeveloped regions remains below 90%^27^. In addition, due to poor living conditions and difficulties accessing healthcare, people living in non-central areas, especially migrants, were more susceptible to HB^1^. The higher incidence of HB in non-central areas suggests that more policy and financial support should be given to promote disease prevention and treatment in these areas.

Some limitations of this study should be noted. First, it is difficult to completely avoid data inaccuracies due to issues with data integrity and quality. Since duplicated reporting of HBV infection is common in China, the results of this study may be overestimated^28^. Second, since not all reporting hospitals performed lgM anti-HBc testing, our analysis was constrained by differences in laboratory capacity. Diagnosis of acute hepatitis B should be based on positive lgM anti-HBc and HBsAg tests along with symptoms related to hepatitis B. However, 10–15% of patients with chronic hepatitis B had IgM anti-HBc, particularly those experiencing acute flare-ups. Consequently, the reported acute hepatitis B incidence was higher than the true incidence. Third, this study lacks estimates for individuals aged 85 years and over. Finally, we were unable to quantify the exposure to some important confounders such as standard of living, sexual behaviors, and blood donation, which may contribute to changes in HBV infection trends.

## Conclusion

In conclusion, this study indicates that the incidence rate of HB is expected to continue decreasing in Guangzhou from 2008 to 2022 due to the widespread use of the HB vaccine and the implementation of the mother-to-child transmission prevention program. However, the chronic HB incidence rate remains relatively high. Age effect, period effect, and cohort effect were observed in the HB epidemic. Adults, especially males, young individuals, and people living in non-central areas were at higher risk of infection. Effective management of chronic HBV infections is still necessary for high-risk populations, and efforts such as screening, vaccination, and antiviral treatment advances should be strengthened to achieve the goal of eliminating viral hepatitis.

### Supplementary Information

Supplementary Information.

## Data Availability

The datasets generated and analyzed in the current study are not publicly available due to the protection of patient privacy but are available from the corresponding authors upon reasonable request.
