# Supplementary material for: Trends and age-period-cohort effect on incidence of hepatitis B from 2008 to 2022 in Guangzhou, China
Source: Sci Rep. 2024 Jun 11;14:13370. doi: 10.1038/s41598-024-63796-0 (PMC11166960; doi:10.1038/s41598-024-63796-0)
Supplement: Supplementary file 1 — Supplementary Information. [file 41598_2024_63796_MOESM1_ESM.pdf]

Trends and age-period-cohort effect on incidence of hepatitis B from 2008 to 2022 in Guangzhou, China

SUPPLEMENTARY MATERIALS

Supplementary Table S1. The reported incidence rate (per 100,000) and cases of Hepatitis B in Guangzhou, China during 2008-2022.

Supplementary Table S2. Annual percent change (APC) and average annual percent change (AAPC) of HB incidence rates using joinpoint regression models in Guangzhou, China during 2008-2022.

Supplementary Figure S1. Long-term trends of age-specific variation of HB incidence in Guangzhou, China during 2008-2022.

Supplementary Figure S2. Long-term trends of period-specific variation of chronic HB incidence in Guangzhou, China during 2008-2022.

Supplementary Figure S3. Long-term trends of period-specific variation of acute HB incidence in Guangzhou, China during 2008-2022.

Supplementary Figure S4. Long-term trends of cohort-specific variation of chronic HB incidence in Guangzhou, China during 2008-2022.

Supplementary Figure S5. Long-term trends of cohort-specific variation of acute HB incidence in Guangzhou, China during 2008-2022.

Supplementary Table S1. The reported incidence rate (per 100,000) and cases of Hepatitis B in Guangzhou, China during 2008-2022.

Variable	Overall			Chronic HB			Acute HB		
	Cases	CIR	ASIR	Cases	ASIR	*P* value	Cases	ASIR	*P* value
**Overall**	327,585	162.18	150.77	309,435	140.19		8,939	4.91	
**Sex**						<0.001			0.036
Male	219,267	208.49	195.70	206,380	181.24		6,346	6.70	
Female	108,318	111.54	102.27	103,055	95.88		2,593	2.96	
**Region**						0.019			0.009
Central areas	120,560	154.34	143.60	116,853	137.89		2,231	3.32	
Non-central areas	207,025	211.58	202.46	192,582	183.43		6,708	8.10	
**Year**									
2008	26,093	239.44	269.71	22,455	231.02		1,253	13.51	
2009	24,857	226.39	257.35	21,561	222.13		1,135	12.35	
2010	23,266	210.47	237.60	21,024	213.36		1,060	11.64	
2011	22,279	175.82	177.94	20,147	159.43		709	6.42	
2012	23,006	181.36	153.55	21,867	145.83		651	4.31	
2013	19,561	153.15	129.39	18,391	121.61		716	4.68	
2014	17,547	137.09	116.43	16,932	112.25		540	3.57	
2015	17,675	137.07	118.13	17,111	114.15		500	3.56	
2016	16,781	125.04	107.41	16,367	104.75		405	2.60	
2017	18,521	132.76	115.17	17,979	111.79		517	3.22	
2018	15,391	106.87	93.57	14,986	91.08		339	2.08	
2019	20,679	139.64	112.64	20,238	110.08		293	1.73	
2020	21,775	143.30	113.09	21,393	111.00		233	1.32	
2021	27,204	146.65	117.07	26,711	114.87		299	1.37	
2022	32,950	177.62	142.46	32,273	139.48		289	1.28	

Note: *HB*: Hepatitis B; *CIR*: Crude incidence rate (per 100,000); *ASIR*: age-standardized incidence rate (per 100,000)

Supplementary Table S2. Annual percent change (APC) and average annual percent change (AAPC) of HB incidence rates using joinpoint regression models in Guangzhou, China during 2008-2022.

Variable	Chronic HB			Acute HB		
	**Segment Year**	**APC (95%CI)**	**AAPC (95%CI)**	**Segment Year**	**APC (95%CI)**	**AAPC (95%CI)**
**Overall**	2008-2016	-10.84(-13.6,-7.99)***	-4.31(-6.51,-2.06)***	2008-2022	-16.87(-19.02,-14.65)***	-16.87(-19.02,-14.65)***
	2016-2022	5.14(0.51,9.99)*				
**Sex**						
Male	2008-2015	-12.87(-16.40,-9.19)***	-5.63(-8.00,-3.21)***	2008-2022	-18.05(-20.33,-15.69)***	-18.05(-20.33,-15.69)***
	2015-2022	2.21(-1.82,6.39)				
Female	2008-2016	-9.40(-12.03,-6.71)***	-2.52(-4.55,-0.44)*	2008-2022	-14.39(-16.77,-11.94)***	-14.39(-16.77,-11.94)***
	2016-2022	7.49(3.30,11.85)**				
**Region**						
Central areas	2008-2017	-14.25(-15.89,-12.57)***	-5.16(-6.91,-3.37)***	2008-2012	-33.89(-47.91,-16.1)**	-16.92(-23.57,-9.67)***
	2017-2022	13.71(8.36,19.32)***		2012-2022	-8.96(-17.02,-0.12)*	
Non-central areas	2008-2020	-7.44(-9.70,-5.13)***	-5.24(-9.47,-0.81)*	2008-2022	-18.43(-20.39,-16.41)***	-18.43(-20.39,-16.41)***
	2020-2022	9.15(-21.69,52.14)				

Note: *HB*: Hepatitis B; *APC*: Annual percent change; *AAPC*: average annual percent change; ****P*<0.001;***P*<0.01;**P*<0.05;

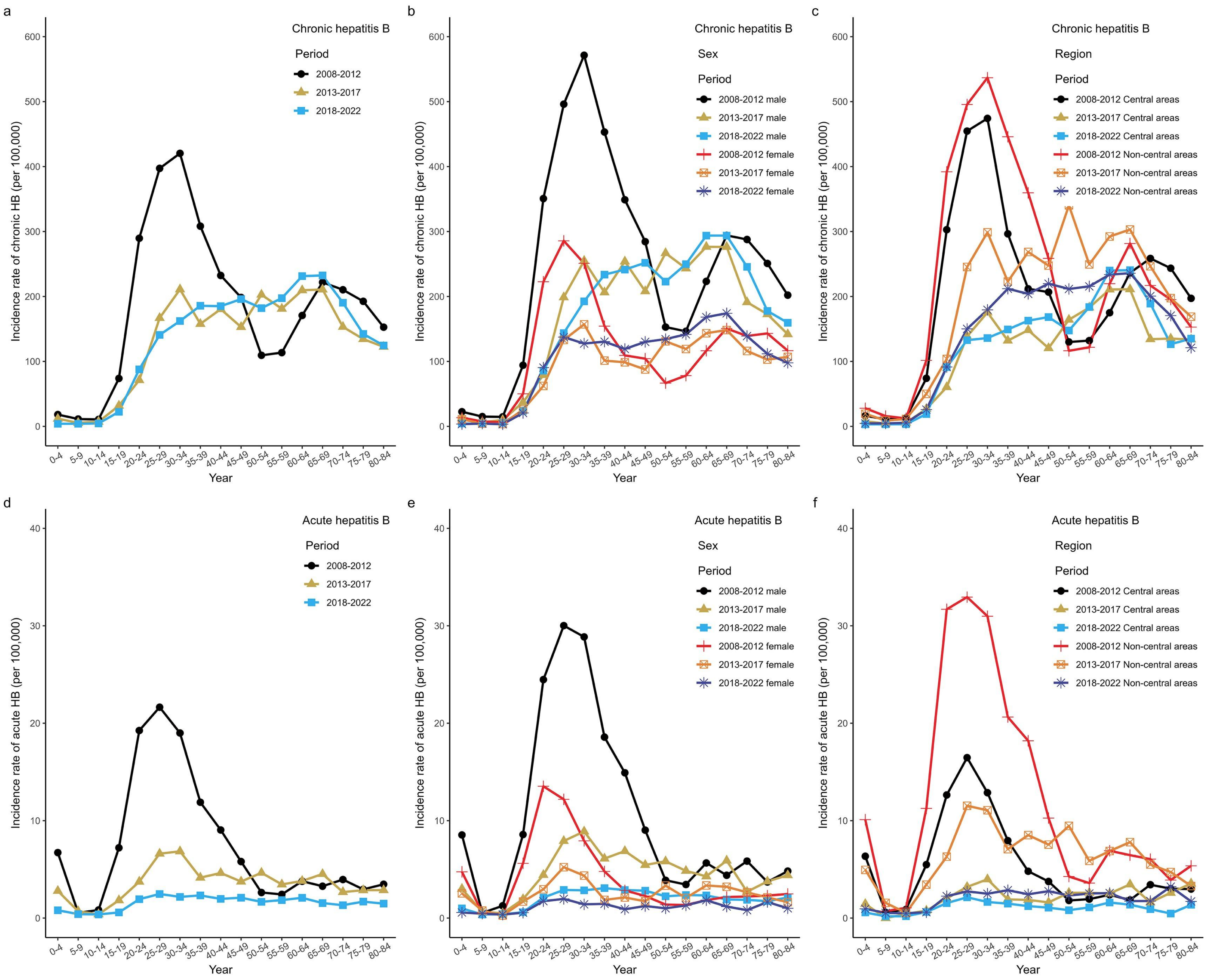

Supplementary Figure S1. Long-term trends of age-specific variation of HB incidence in Guangzhou, China during 2008-2022. (a) Overall for chronic HB; (b) Grouped by sex for chronic HB; (c) Grouped by region for chronic HB; (d) Overall for acute HB; (e) Grouped by sex for acute HB; (f) Grouped by region for acute HB.

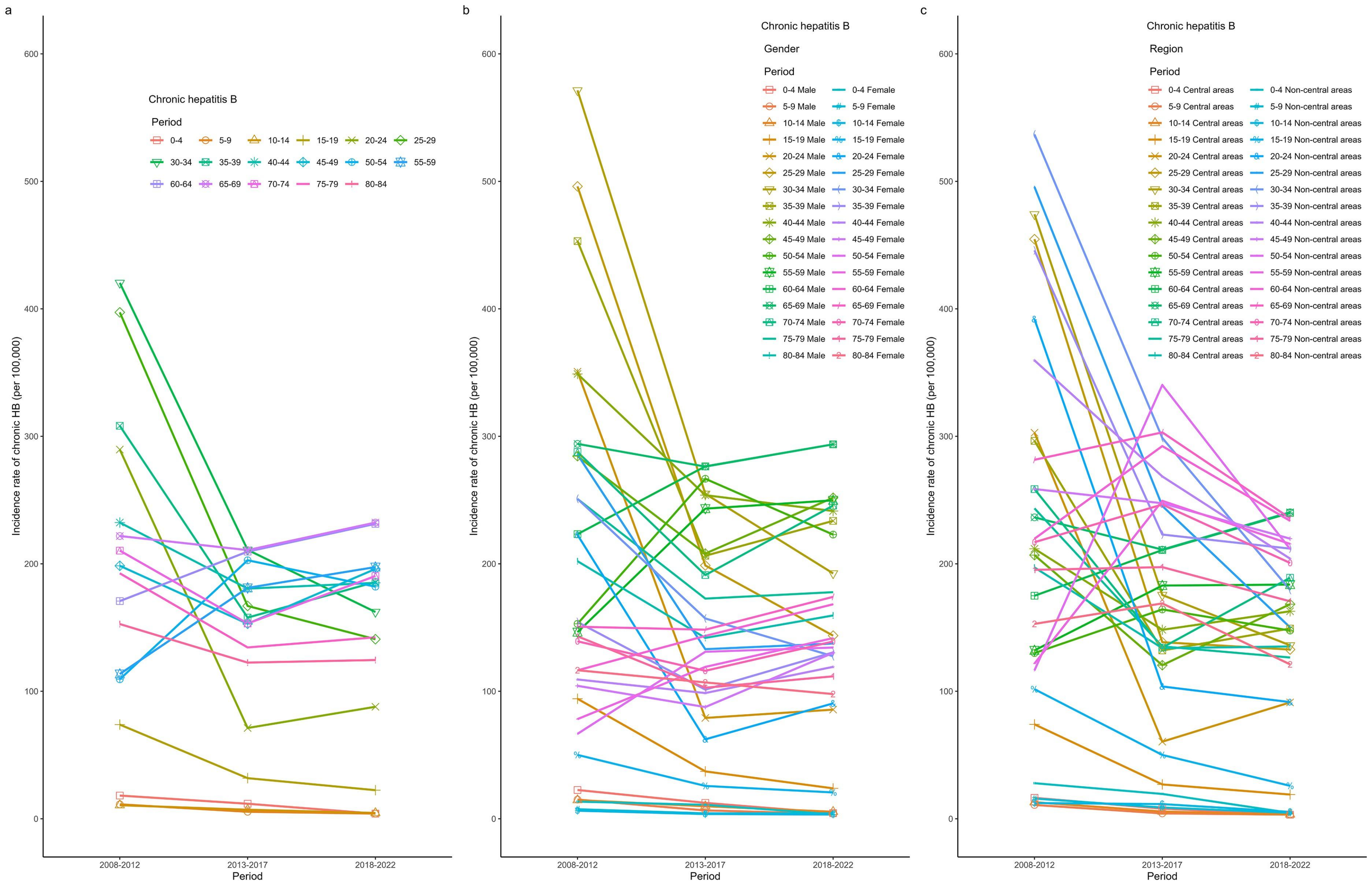

Supplementary Figure S2. Long-term trends of period-specific variation of chronic HB incidence in Guangzhou, China during 2008-2022. (a) Overall; (b) Grouped by sex; (c) Grouped by region;

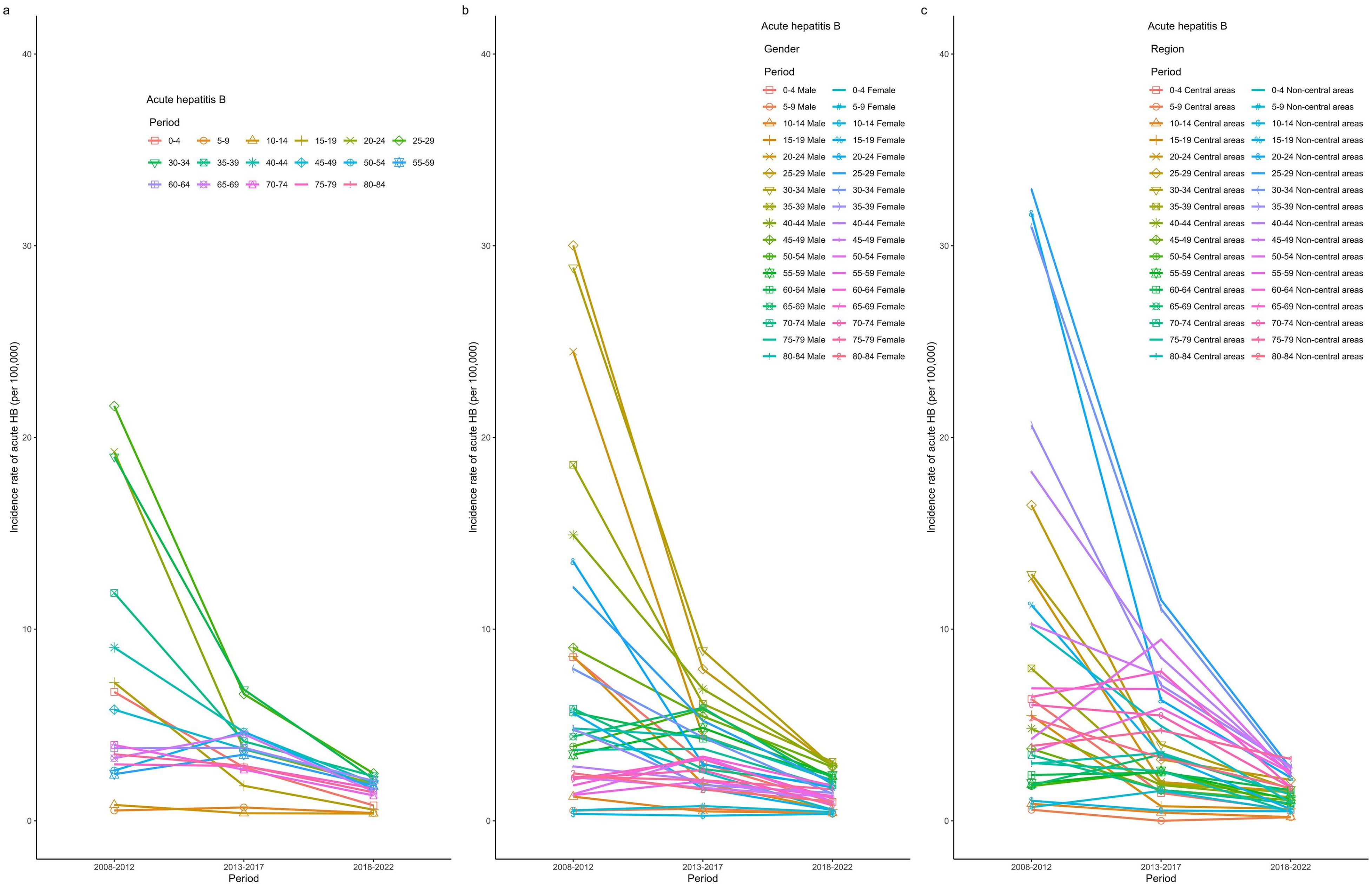

Supplementary Figure S3. Long-term trends of period-specific variation of acute HB incidence in Guangzhou, China during 2008-2022. (a) Overall; (b) Grouped by sex; (c) Grouped by region.

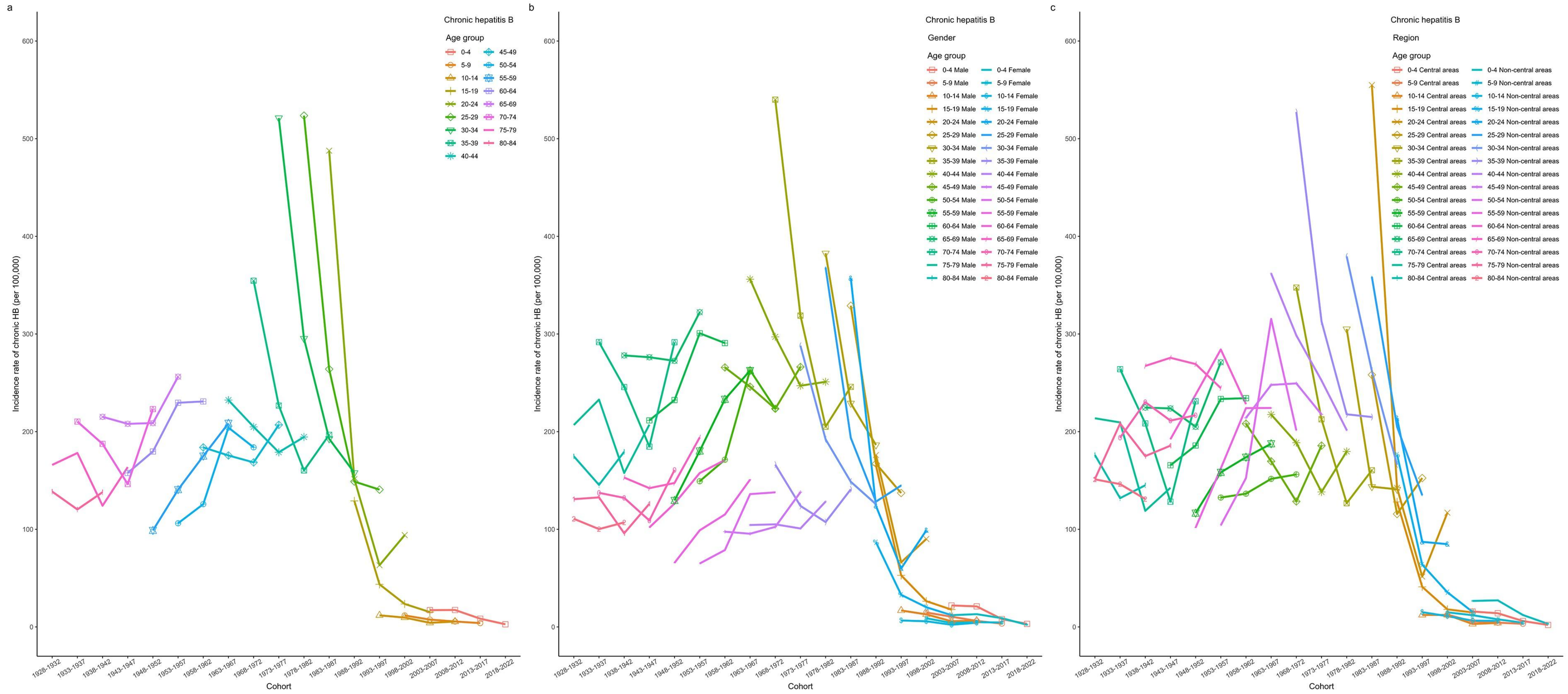

Supplementary Figure S4. Long-term trends of cohort-specific variation of chronic HB incidence in Guangzhou, China during 2008-2022. (a) Overall; (b) Grouped by sex; (c) Grouped by region.

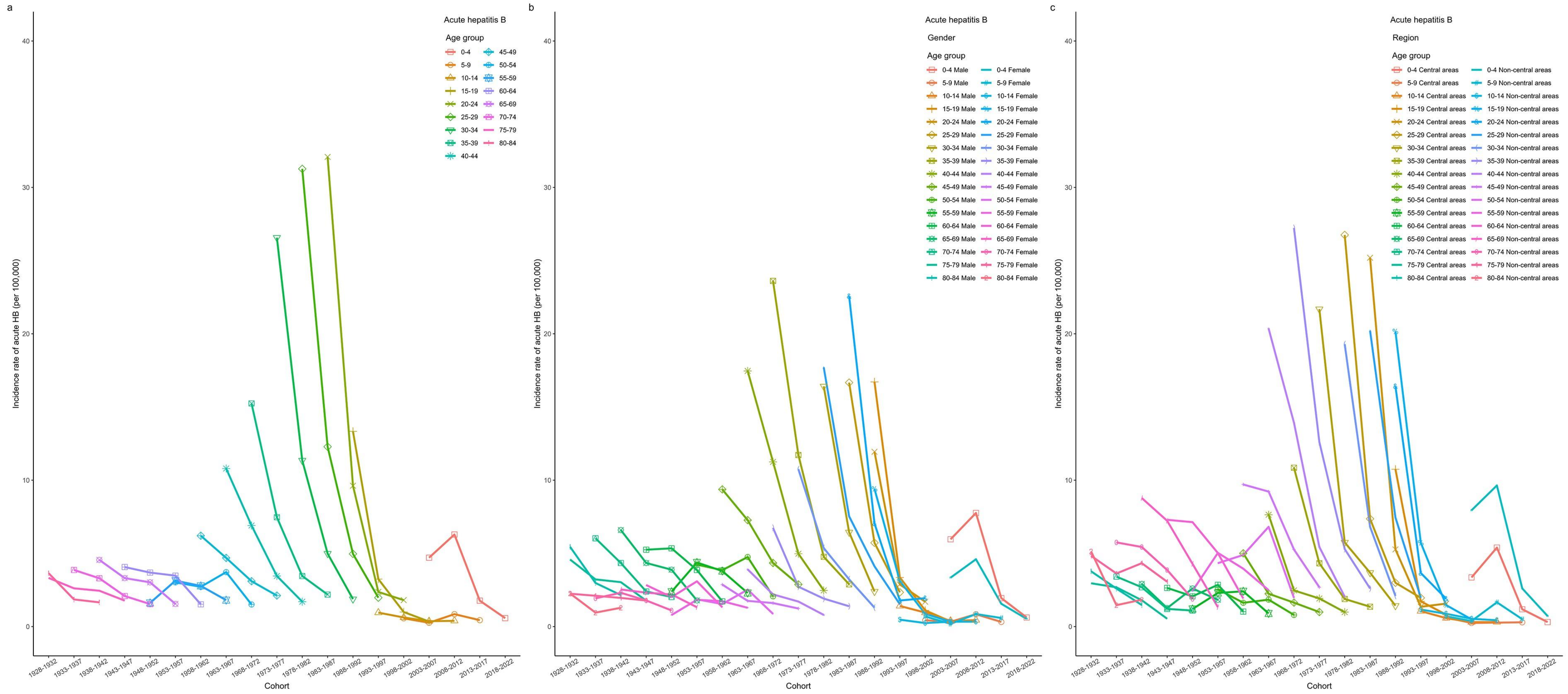

Supplementary Figure S5. Long-term trends of cohort-specific variation of acute HB incidence in Guangzhou, China during 2008-2022. (a) Overall; (b) Grouped by sex; (c) Grouped by region.
